# Effects of empagliflozin on reproductive system in men without diabetes

**DOI:** 10.1038/s41598-024-64684-3

**Published:** 2024-06-14

**Authors:** Christophe Kosinski, Georgios E. Papadakis, Olivier Salamin, Tiia Kuuranne, Raul Nicoli, Nelly Pitteloud, Anne Zanchi

**Affiliations:** 1 Service of Endocrinology, Diabetes and Metabolism, Lausanne University Hospital and University of Lausanne, Lausanne, Switzerland; 2 Service of Endocrinology, Diabetes, Nutrition and Therapeutic Patient Education, Geneva University Hospitals, Geneva, Switzerland; 3 Service of Nephrology, Lausanne University Hospital and University of Lausanne, Lausanne, Switzerland; 4 Swiss Laboratory for Doping Analyses, University Center of Legal Medicine, Lausanne and Geneva, Lausanne University Hospital and University of Lausanne, Lausanne, Switzerland

**Keywords:** Reproductive system, SGLT2 inhibitors, Empagliflozin, Testosterone, Clinical trials, Endocrinology, Endocrine reproductive disorders

## Abstract

Sodium-glucose cotransporter (SGLT) 2 inhibition is a well-known target for the treatment of type 2 diabetes, renal disease and chronic heart failure. The protein SGLT2 is encoded by SLC5A2 (Solute Carrier Family 5 Member 2), which is highly expressed in renal cortex, but also in the testes where glucose uptake may be essential for spermatogenesis and androgen synthesis. We postulated that in healthy males, SGLT2 inhibitor therapy may affect gonadal function. We examined the impact on gonadal and steroid hormones in a post-hoc analysis of a double-blind, randomized, placebo-controlled research including 26 healthy males who were given either placebo or empagliflozin 10 mg once daily for four weeks. After one month of empagliflozin, there were no discernible changes in androgen, pituitary gonadotropin hormones, or inhibin B. Regardless of BMI category, the administration of empagliflozin, a highly selective SGLT2 inhibitor, did not alter serum androgen levels in men without diabetes. While SGLT2 is present in the testes, its inhibition does not seem to affect testosterone production in Leydig cells nor inhibin B secretion by the Sertoli cells.

## Introduction

Sodium-glucose co-transporter 2 (SGLT2) inhibitors, an established therapeutic class for the management of type 2 diabetes, symptomatic heart failure and proteinuric kidney disease, primarily target the SGLT2 co-transporter found in the proximal tubule of the kidney^1^. SGLT2 is encoded by *SLC5A2* (Solute Carrier Family 5 Member 2), which is highly expressed in the renal cortex^2,3^. In addition, this gene is also expressed in the testes^2,3^, potentially concentrated in the seminiferous tubules, where the uptake of glucose might be essential for spermatogenesis*.* Data regarding the effect of SGLT2 inhibitors on male reproductive function are very scarce and conflicting. In the streptozotocin animal model of diabetes, therapy with SGLT2 inhibitors induced apoptosis in the seminiferous tubules and sperm morphological damage^4^. Conversely, in streptozotocin-induced diabetic male rats, a 8-weeks treatment with SGLT2 inhibitor empagliflozin improved serum levels of LH, testosterone, insulin, leptin, and the expression of kisspeptin in the testes tissues^5^. Moreover, in leptin receptor-deficient diabetic mice, blocking SGLT2 improved the health of seminiferous tubules and increased sperm concentration and motility^6^. In humans, available data are limited to one retrospective study in men with uncontrolled type 2 diabetes, who received different combinations of antidiabetic regimens, including a subgroup of 16 patients who received metformin combined with the SGLT2 inhibitor dapagliflozin. An improvement of functional hypogonadism was observed, but the positive effect was mainly driven by weight loss and not the treatment group per se^7^.

Given that these treatments are now increasingly prescribed in individuals with and without diabetes, we assessed whether treatment with SGLT2 inhibitor empagliflozin could interfere with gonadal function in men without diabetes.

## Material and methods

This was a post-hoc analysis of a double‐blind, randomized, placebo‐controlled study. Details about the study have been published elsewhere^8,9^. The primary study aimed to determine whether empagliflozin alters renal oxygenation and to assess its metabolic, renal and hemodynamic effects in non-diabetic subjects; it concluded that empagliflozin causes glucosuria and transient natriuresis, reduces blood pressure, but does not affect renal oxygenation. A 2:1 randomization (empagliflozin:placebo) was used to compensate for a potentially higher drop-out rate in the empagliflozin group due to adverse effects. For the current analysis, we used only data of male participants with complete data about gonadal analysis.

The ethics commission of the Canton de Vaud, Switzerland, approved this monocentric research project, which was carried out in accordance with the principles of the Declaration of Helsinki. A written informed consent was obtained from all participants.

Data of 26 healthy men were analyzed, of which 8 were randomized to the placebo and 18 to the once-daily 10mg empagliflozin group, during 1 month. Serum samples were analyzed using an ultra-high-performance LC (UHPLC)–MS/MS method at the Swiss Laboratory for Doping Analyses in Lausanne, Switzerland, for quantification of steroid hormones including total testosterone (TT) and dihydrotestosterone (DHT)^10^. TT and sex hormone-binding globulin were used to calculate free testosterone (FT) with Vermeulen formulation^11^. Serum inhibin B, luteinizing hormone (LH) and follicle-stimulating hormone (FSH) were measured in the central laboratory of the Lausanne University Hospital using automated immunoassays (Architect, Abbott, USA). Blood sampling was performed in the morning in a fasting state.

Statistical analysis was performed using STATA version 16.0 (StataCorp, College Station, TX). Quantitative variables are expressed as mean ± standard deviation. Qualitative variables are expressed as number and percentage. Paired student's t-test was used to evaluate the effects of empagliflozin and placebo at one month.

## Results

In the 26 male subjects included in the clinical trial, mean age was 31.7 ± 7.7 years and body mass index was 29.3 ± 4.6 kg/m^2^. All weight categories were included: 6 normal weight (body mass index, BMI, 18–24.9 kg/m^2^), 9 overweight (BMI 25–29.9 kg/m^2^), 11 obese (BMI ≥ 30 kg/m^2^). Other clinical characteristics are detailed in Zanchi et al.^9^. All participants had normal TT (> 10.4 nmol/L), except one (9.82 nmol/L), as well as LH and FSH levels (< 9 U/l and < 12 U/l, respectively) at baseline.

**Figure 1:**
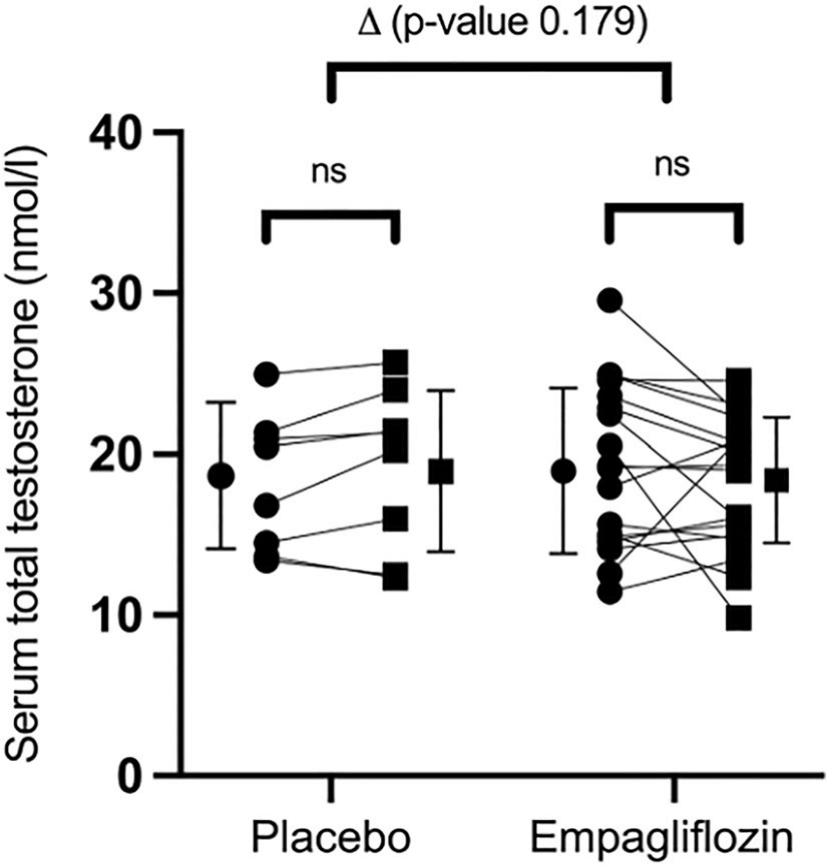
Effect of empagliflozin on serum total testosterone. (● = baseline; ■ = after 1 month).

After one month therapy, no significant changes were observed in serum TT (− 1.2 ± 4.2 nmol/L with empagliflozin, 0.9 ± 1.6 nmol/L with placebo; difference between group (∆): − 2.1 ± 1.5 nmol/L; all *p* > 0.1) (FIGURE 1), DHT (0.06 ± 0.39 nmol/L with empagliflozin, 0.03 ± 0.19 nmol/L with placebo; ∆: 0.03 ± 0.15 nmol/L; all *p* > 0.1) and FT (− 0.02 ± 0.13 nmol/L with empagliflozin, 0.01 ± 0.05 nmol/L with placebo ∆: 0.04 ± 0.05 nmol/L; all *p* > 0.1) (supplementary appendix).

**Figure 2:**
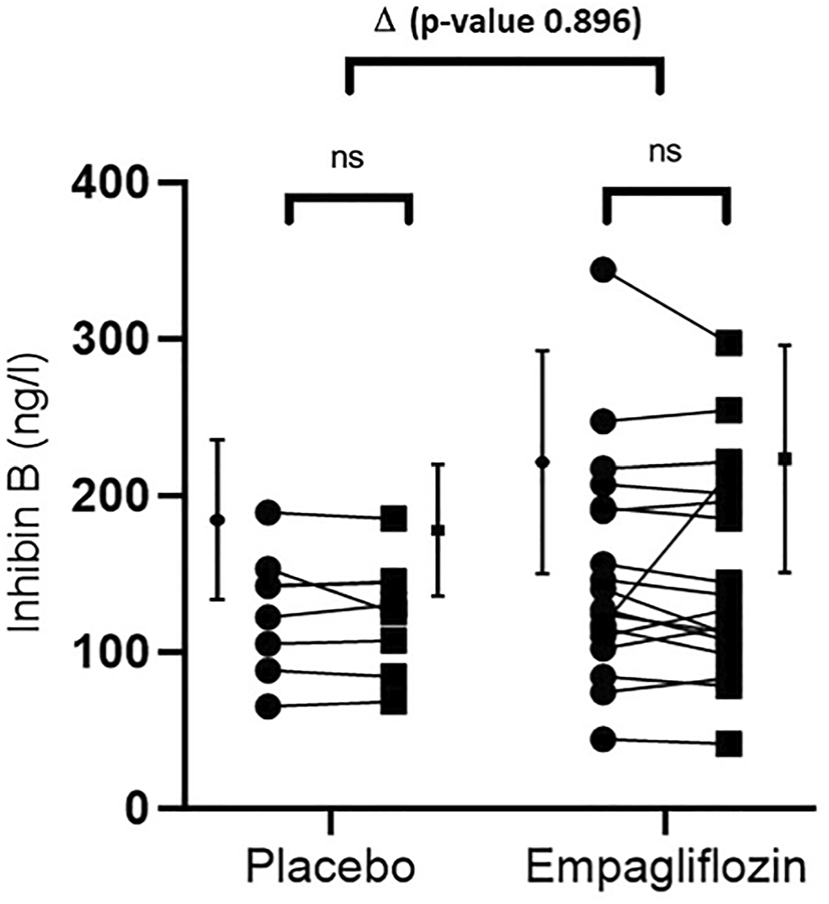
Effect of empagliflozin on serum inhibin B. (● = baseline; ■ = after 1 month).

Furthermore, no significant changes were observed in inhibin B levels (FIGURE 2), LH or FSH (all p > 0.1) (supplementary appendix).

No significant variations in weight were observed after one month of empagliflozin, and there was no effect of baseline weight on testosterone changes (data not shown). There were no changes in steroid profile (androstenedione, 17α-OH-Progesterone, dehydroepiandrosterone (DHEA), corticosterone, cortisol, deoxycorticosterone, 11-Deoxycortisol) after one month of empagliflozin therapy (supplementary appendix).

## Discussion

The use of a highly selective SGLT2 inhibitor, empagliflozin, in healthy men did not alter serum testosterone levels, irrespective of baseline BMI category. As SGLT2 receptors are expressed in the seminiferous tubules, inhibin B, which is an established marker of Sertoli cell and spermatogenesis function, was also measured^12^. Since no significant effect of empagliflozin was observed on these levels, an effect on spermatogenesis in men without diabetes is unlikely. This was also further confirmed by the absence of any change in FSH levels, which promptly increase in response to seminiferous tubule damage.

To our knowledge, no study has assessed the potential effect of SGLT2 inhibitors on gonadal function in men without diabetes. As this therapeutic class is being extensively and increasingly used, it was important to demonstrate the absence of any negative effect. Indeed a reduced glucose intake at testicular level, the source of energy, could have an effect on spermatogenesis^13^. Treatment with an SGLT2 inhibitor also had no effect on the other steroid sex hormones such as DHEA-S and androstenedione, as would be expected given the location of their production mainly in the adrenal cortex.

However, certain limitations must be acknowledged. The number of cases is modest, but there was no trend suggesting an effect that might have become significant with a larger number. The duration of treatment was short, although the effect on the sodium glucose transporter is immediate after the first pill. Finally, these results cannot be translated to men with type 2 diabetes as SGLT2 inhibition could lead to improve serum testosterone levels secondary to weight loss. The absence of weight loss in the current study allows, thus, to dissect the direct action of SGLT2 inhibition on testicular function, providing reassuring data for their reproductive safety.

In conclusion, although SGLT2 is expressed in the testis and contrary to animal data, the use of a highly selective SGLT2 inhibitor, empagliflozin, does not affect serum testosterone or inhibin levels in men without diabetes. Any beneficial effects previously shown in diabetic populations seem to not reflect direct gonadal effects but to be mediated by the SGLT2 inhibitor-associated weight loss.

### Supplementary Information

Supplementary Table 1.

## Data Availability

The data will be shared on reasonable request to the corresponding or the senior author.
