# Supplementary material for: Effects of empagliflozin on reproductive system in men without diabetes
Source: Sci Rep. 2024 Jun 14;14:13802. doi: 10.1038/s41598-024-64684-3 (PMC11178909; doi:10.1038/s41598-024-64684-3)
Supplement: Supplementary file 1 — Supplementary Table 1. [file 41598_2024_64684_MOESM1_ESM.docx]

**Supplementary table 1. Effects on sexual hormones and steroid profil.**

| **Variables** | **Empagliflozin (n=16)** | | **Placebo (n=8)** | |  |
| --- | --- | --- | --- | --- | --- |
|  | *Baseline* | *1 month* | *Baseline* | *1 month* | Δ *(p-value)* |
| Total testosterone (nmol/L) | 19.33±5.1 | 18.12±4.1 | 18.24±4.3 | 19.18±5.0 | -2.14±1.55 *(0.18)* |
| DHT (nmol/L) | 1.34±0.46 | 1.40±0.54 | 1.50±0.45 | 1.52±0.60 | 0.03±0.15 *(0.83)* |
| Free testosterone (nmol/L) | 0.48±0.13 | 0.45±0.11 | 0.44±0.10 | 0.45±0.13 | -0.04±0.05 *(0.47)* |
| Bioactive testosterone (nmol/L) | 10.56±2.65 | 11.17±3.01 | 10.24±2.4 | 10.50±2.96 | -0.88±1.15 *(0.45)* |
| SHBG (nmol/L) | 26.39±13.06 | 25.52±13.14 | 26.68±5.75 | 28.64±7.53 | -2.84±1.71 *(0.11)* |
| Free androgen index | 87.90±44.10 | 86.39±38.77 | 69.03±13.47 | 68.43±17.77 | -0.90±10.53 *(0.93)* |
| Inhibin B (ng/ml) | 152.00±29.02 | 151.17±67.93 | 125.75±39.34 | 123.50±37.07 | 1.42±10.69 *(0.89)* |
| LH (U/L) | 5.68±1.96 | 5.98±2.78 | 5.62±2.36 | 5.31±2.17 | 0.61±1.47 *(0.68)* |
| FSH (U/L) | 3.56±1.44 | 4.69±2.89 | 3.40±0.76 | 4.35±1.61 | 0.19±1.39 *(0.89)* |
| Steroid profile |  |  |  |  |  |
| Androstenedione (nmol/L) | 2.52±1.40 | 2.35±1.44 | 2.07±0.39 | 1.87±0.23 | 0.03±0.33 *(0.93)* |
| 17α-OH-Progesterone (nmol/L) | 2.89±1.20 | 2.55±0.97 | 2.97±1.67 | 3.06±1.50 | -0.43±0.32 *(0.19)* |
| DHEA (nmol/L) | 17.27±12.21 | 15.89±12.98 | 13.64±6.33 | 11.03±3.13 | 1.23±2.03 *(0.55)* |
| Corticosterone (nmol/L) | 7.61±11.66 | 7.64±8.61 | 6.86±5.83 | 3.88±2.70 | 3.01±1.71 *(0.09)* |
| Cortisol (nmol/L) | 264.53±28.51 | 266.68±100.21 | 261.54±50.81 | 214.56±50.81* | 49.13±39.02 *(0.22)* |
| Deoxycorticosterone (nmol/L) | 0.14±0.11 | 0.14±0.09 | 0.12±0.03 | 0.10±0.03# | 0.01±0.02 *(0.51)* |
| 11-Deoxycortisol (nmol/L) | 0.055±0.65 | 0.57±0.66 | 0.47±0.22 | 0.41±0.24 | 0.08±0.08 *(0.34)* |
| Abbreviations: DHT, dihydrotestosterone; SHBG, sex hormone binding globulin; LH, luteinizing hormone; FSH, follicle-stimulating hormone; DHEA, dehydroepiandrosterone.  Δ: between group changes comparison.  All data are presented as mean±SD, except Δ, as mean±SE.  Changes at 1 month are all p>0.05, except * (p=0.0460) and # (p=0.0179) | | | | | |
